# Fibrolamellar Carcinoma: 2012 Update

**DOI:** 10.6064/2012/743790

**Published:** 2012-09-23

**Authors:** Michael Torbenson

**Affiliations:** Department of Pathology, The Johns Hopkins University School of Medicine, Room B314, 1503 E. Jefferson, Bond Street Building, Baltimore, MD 21231, USA

## Abstract

Fibrolamellar carcinomas are a unique type of primary liver cancer. They occur most commonly in children and young adults. Their etiology remains a mystery, as they are not associated with chronic liver disease. Fibrolamellar carcinomas are not indolent tumors, but have an overall better prognosis than typical hepatocellular carcinomas, in part because of the younger age at presentation and the lack of cirrhosis. The most important prognostic feature is whether the tumor is resectable. Histologically, the tumor is made up of large cells that contain abundant mitochondria. The nuclei of the tumor cells have prominent nucleoli. The tumor cells induce the formation of extensive intratumoral fibrosis, which often grows in parallel, or lamellar bands. The tumor cells clearly show hepatocellular features but are also unique in showing both biliary and neuroendocrine differentiation. The uniqueness of fibrolamellar carcinoma extends to their molecular findings. While the genetic abnormalities that lead to fibrolamellar carcinomas are not yet known, studies have shown that they lack mutations in the genes most commonly mutated in typical hepatocellular carcinoma (*TP53* and *CTNNB1*). In this paper, the clinical, pathological, and basic science literature on fibrolamellar carcinoma is comprehensively reviewed. Key areas of needed research are also discussed.

## 1. Introduction

Fibrolamellar carcinoma (FLC) was first described as a unique entity in 1956 by Edmondson [1], who described it as a unique form of primary hepatocellular carcinoma. Craig et al. further developed the striking clinical predilection for younger individuals and the characteristic histological features in 1980 [2]. This 1980 study was also the first to use the term “fibrolamellar carcinoma.” Subsequent case reports and cases series have confirmed and expanded the unique clinical and pathologic features of FLC. Major review articles on the clinical, pathological, and biological findings have been published in 2007 [3], 2009 [4], and 2011 [5]. 

While earlier editions of the series WHO Classification of Tumors recognized FLC as a unique histological pattern, it was not until the 2010 edition [6] that FLC was given its own WHO classification number. This was an important step because FLC is unique at the clinical, histological, and biological levels and they should be analyzed separately from typical hepatocellular carcinomas in all scientific studies.

## 2. Clinical Findings

### 2.1. Presentation

FLC generally present with vague, nonspecific clinical signs and symptoms, often which include elements of abdominal pain, weight loss, and malaise [2, 7–11]. Overall, the most common physical finding is an abdominal mass or hepatomegaly [9–11]. However, a wide variety of unusual presentations have also been described (Table 1). These include several reports of biliary obstruction secondary to direct tumor growth into the biliary tree or to compression by metastatic deposits in hilar lymph nodes. In fact, biliary obstruction is probably even more common than reported, as dilated intrahepatic bile ducts are found in 40% of cases by imaging studies [12]. Gynecomastia has also been reported by several authors [13–16]. 

### 2.2. Serum Findings

Serum levels of aspartate aminotransferase (AST) and alanine aminotransferase (ALT) can be normal [11] or mildly elevated [2]. Alkaline phosphatase levels may be elevated [10, 32, 34], occasionally with levels greater than 1000 IU/mL [23], findings that most likely reflect growth into the biliary tree or obstruction of the biliary tree. Alpha-fetoprotein (AFP) levels are typically normal [35]. There is a subset of approximately 5–10% of reported cases in the literature that have serum AFP elevations in the 200 ng/mL or greater range. For example, in one of the larger series to date, 7% of the FLC had AFP levels greater than 200 ng/mL [36]. As a second example, 27% of cases in another study had elevated serum AFP levels [10]. However, overall it seems highly likely that a substantial proportion of the reported FLC cases with elevated serum AFP are misdiagnosed and should have been classified as typical hepatocellular carcinoma. While the histological features of FLC, as described in the Histology section, are quite distinctive, misclassification of typical hepatocellular carcinoma as FLC is a persistent and significant problem when evaluating the published literature. 

Other serum findings in patients with FLC include elevated transcobalamin I levels [7, 37, 38], transcobalamin 2 [7], and vitamin B12 binding capacity [7, 19, 23, 37, 39, 40]. Transcobalamin I is also known as haptocorrin and is a glycoprotein that in normal physiology is made largely in the salivary glands, from where it is secreted into the digestive tract and protects vitamin B12 (also known as cobalamin) from degradation in the stomach. The reasons for the elevated B12 binding capacity and the elevated transcobalamin levels in FLC are not clear, but have been reported by multiple groups. Increased vitamin B12 binding capacity is more commonly seen in FLC than typical hepatocellular carcinoma [39] but is not exclusive to FLC. Serum transcobalamin I or haptocorrin levels can also be elevated in cases of typical hepatocellular carcinoma [41] and, in these cases, appear to be produced by tumor cells or have increased serum uptake by the tumor cells [42]. 

Other elevated serum proteins include fibrinogen [19]. Interestingly, fibrinogen can also be found within the “pale bodies” of FLC tumor cells (see below). Serum neurotensin levels can also be elevated in about 25% of FLC cases [43]. In one study on neurotensin, 20 patients with liver cancer were studied for serum neurotensin levels: five patients had elevated levels and four were diagnosed with FLC [44]. Finally, PIVKA-II (protein induced by vitamin K absence/antagonist-II), perhaps better known as Des-gamma carboxyprothrombin or DCP, was elevated in all cases of FLC in a USA-based study of 41 cases [45] and in approximately 70% of the cases in a report from Japan [19]. This protein is an abnormal form of the coagulation protein, prothrombin, and is also frequently elevated in typical HCC [46]. 

### 2.3. Demographics

One of the most distinctive and well-recognized features of FLC is its occurrence in younger individuals. To get a stronger sense of the age distribution in FLC, the age at diagnosis of FLC was extracted from the literature of 275 cases where individual ages were provided (data extracted from reports in the references list). It is recognized that a proportion of these cases were incorrectly diagnosed as FLC, and a few cases of obvious misdiagnosis were excluded. While this is a limitation, the data nonetheless provides the most comprehensive survey of age at diagnosis available for FLC. Based on these 275 cases, the age at presentation shows a unimodal distribution that rises sharply in the late teens and peaks in the early twenties, with an overall average age of 25.6 ± 12.6 years (mean ± standard deviation) and a median of 22 years and a mode of 21 years. In contrast, an average age of 39 ± 20 years was reported for 68 individuals from the Surveillance, Epidemiology, and End Results (SEER) database [47]. These latter results, while still younger than the average presenting age for typical hepatocellular carcinoma, are significantly older than the aggregate of cases reported in the literature and suggest that many of the cases in the SEER database are misclassified. Nonetheless, the strong pattern remains: FLC occurs at significantly younger ages than typical hepatocellular carcinoma, which has an average age at presentation of 65 years [47]. 

Based on extracted age information from 275 cases in the literature, FLC strongly clusters in the young with 50% of all cases occurring between the ages of 17 and 30 and 80% within the years 10 to 35. The youngest age reported in the literature is 7 months [48], but this case is such an exception that the diagnosis is strongly suspected. Likewise, the published images in another reported case of FLC in a 4-month-old infant do not strongly support the diagnosis of FLC [49]. However, FLC has been reported by several groups as early as 4 to 5 years of age [2, 50, 51]. Of note, FLC can occur in older individuals, including rare cases in those older than 60 years. 

Overall, about 20% of typical hepatocellular carcinomas arise in noncirrhotic livers. Interestingly, these carcinomas share some broad overall similarities with FLC: they are often reported to present at a younger age [52], do not have as a strong of a skewed male : female ratio as hepatocellular carcinomas arising in cirrhotic livers [52], and frequently recur after surgery with curative intent [52]. 

### 2.4. Gender and Ethnicity

No strong gender predilection is apparent for FLC [8, 11, 45, 47]. Based on the SEER database, there appears to be a racial/ethnic predilection for Caucasians [47]. However, FLC has been reported from a wide variety of geographical locations including Mexico [53], Saudi Arabia [33], South Africa [54, 55], Japan [56], Taiwan [57, 58], Mainland China [59], Republic of Korea [60], Thailand [32], India [28, 61], and Europe [62], and the extent of the racial/ethnic bias remains poorly defined.

### 2.5. Frequency

Overall, FLC comprises approximately 5% of all hepatocellular carcinomas, but the exact proportion varies between less than 1% and 8% depending on study design and the patient population (Table 2).

Because of the well-known predilection of FLC for young individuals, there is a common misconception that FLC is the most frequent form of liver cancer in children and young adults. However, the most common form of liver cancer in children and young adults is in fact typical hepatocellular carcinoma, which still accounts for between 60% and 80% of liver cancers in this age group [48, 50, 67, 68]. 

### 2.6. Prognosis

The prognosis of FLC has been studied for many years. Earlier studies were often characterized by comparing FLC prognosis to that of typical hepatocellular carcinoma. Since most individuals with typical hepatocellular carcinoma are considerably older and have liver cirrhosis, as well as other comorbid conditions, patients with FLC appeared to do significantly better. However, in these cases, the better prognosis observed for FLC is in part due to the lack of cirrhosis and other comorbid conditions. Cirrhosis, for example, is a well-recognized independent risk factor for mortality. Thus, when FLC is compared only to those hepatocellular carcinomas that arise in the background of noncirrhotic livers, the survival advantage is less apparent, with no survival benefit seen in some case series [69], although a survival benefit was still evident in others [45, 67, 70]. 

### 2.7. Clinical and Pathology Prognostic Factors

Resectability is one of the most important prognostic features for FLC [36, 47, 48, 53]. For example, in one large study, the median 5-year survival for those who underwent resection was 76% with a median overall survival of 112 months [36]. In contrast, the median 5-year survival for those with unresectable disease was 0% with an overall median survival of 12 months [36]. Another study of FLC patients with unresectable liver disease due to regional node disease found a similar median survival of 14 months [71]. 

Other reported prognostic factors include gender [47], age at presentation [47, 53], lymph node [36, 45] or other metastatic disease [45] at the time of presentation, tumor stage [11, 45, 47], vascular invasion [45], absence of elevated liver enzymes [53], and absence of large vessel invasion or thrombosis [53]. However, most of these additional prognostic findings have not been well validated across multiple studies.

### 2.8. Treatment

As noted previously, surgical resection is a key first line treatment. However, even in those patients with resectable diseases, subsequent recurrent liver or metastatic tumor is seen in more than half of the cases, with a reported range from 36% to 100% [7, 36]. The median time to recurrence tends to be short, ranging from 10 to 33 months, though later recurrences have also been described. Aggressive surgical resection of metastatic disease, when possible, can be of benefit [7, 11].

In terms of systemic chemotherapy, data is limited but there have been reports of partial response to platinum-based therapy [7]. Overall, FLC does not respond better than typical HCC to cisplatin, vincristine, and 5-FU-based chemotherapy [48]. Others have found no clear benefit for chemotherapy in individuals as adjuvant therapy for FLC [7, 8, 45].

### 2.9. Section Summary and Key Remaining Questions

 In sum, FLC occurs in younger individuals with no strong gender bias. FLC has been reported from many different ethnic backgrounds, but the overall current findings suggest a possible Caucasian predominance. FLC typically presents with advanced stage disease, is locally aggressive with a propensity to metastasize, and has a high relapse following resection. Cure rate is very low. Treatment remains focused on surgical resection with aggressive management of surgical disease, with either no or modest long-term benefit from systemic chemotherapy. 

Key questions that remain to be answered or substantially clarified include the following: *Question *1. One of the critical points to future progress is to ensure that reported cases of FLC actually meet the histological requirements for this diagnosis. It is a significant problem because studies that inadvertently include cases of typical hepatocellular carcinoma in studies of FLC confuse the literature and confound our ability to make future progress. As discussed below, there are now several histological immunostains that can be used to confirm the diagnosis of FLC and all authors should be strongly encouraged to use them. *Question *2. Is there truly a Caucasian predominance? If so, can a more-specific-at-risk Caucasian population be identified? *Question *3. Are there any regional risk factors for FLC that are not explained by ethnicity, such as urban versus rural, etc.? and as a related question, are there geographic or temporal clustering to cases of FLC? *Question *4. What is the best currently available chemotherapeutic agent useful in patients with FLC? 

## 3. Tumor Findings

### 3.1. Gross Findings

Grossly, FLCs are yellow to pale tan in color and their consistency can range from soft to firm and hard. A central scar is found in 60–70% of cases [10, 72]. The tumors tend to be more common in the left lobe of the liver [2, 12], but frequently involve both lobes [72]. At the time of resection, the average tumor diameter is large, ranging between 9 and 14 cm in greatest dimension [8, 10, 12, 36, 45]. In 80–90% of cases, a single large tumor is present [8, 12, 36, 45, 66]. Occasionally, the tumors contain small foci of necrosis as well as areas of hemorrhage [72]. Gross vascular invasion is seen in up to 25% of cases [45].

The background livers are noncirrhotic. While several cases of FLC in the setting of cirrhosis were included in the earliest descriptions of FLC [2] and have been occasionally reported subsequently [45], the diagnosis of FLC in the setting of cirrhosis should be carefully considered, as it seems likely that a large proportion of the FLCs reported in this context are misclassified.

### 3.2. Histology Findings

**Figure 1:**
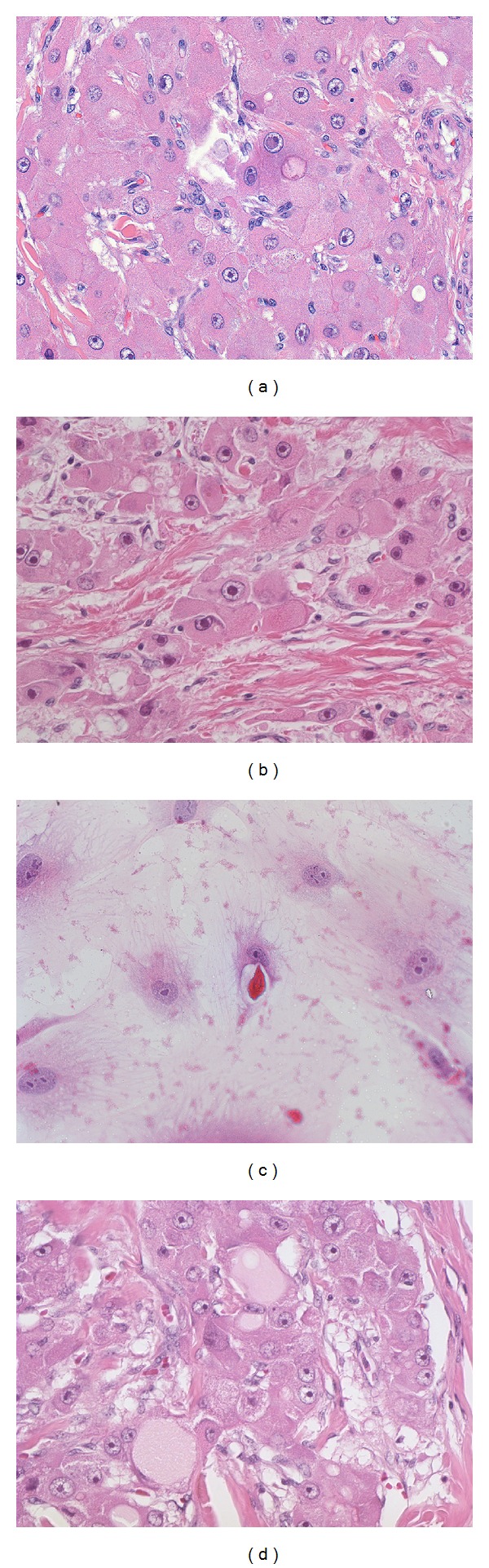
(a) Original magnification 160X. Fibrolamellar carcinomas are composed of large eosinophilic tumor cells with large vesiculated nuclei and large red nucleoli. (b) Original magnification 100X. Intratumoral fibrosis is a typical finding in fibrolamellar carcinoma. (c) Original magnification 160X. When FLC tumors grow in cell culture, they retain their large nucleoli and abundant cytoplasm. The nucleolus is often split into several larger subunits. (d) Original magnification 160X. Pale bodies are seen as large cytoplasmic inclusions with distinct borders and typically a “pale” grey color.

The tumor is made up of large polygonal cells with abundant eosinophilic cytoplasm, large vesiculated nuclei, and large nucleoli (Figures 1(a) and 1(b)). These three cytological findings in conjunction with the lamellar fibrosis Figure 1(b) are the defining features of FLC. FLC cells grown in culture retain their prominent nucleolus, though it may be found in several smaller structures that are likely distributed amongst different chromosomes (Figure 1(c)). The eosinophilic appearance of the tumor cells is a result of mitochondrial proliferation (see Ultrastructure section). In approximately 1/2 of cases, the tumor cells can have round amphophilic cytoplasmic inclusions, termed “pale bodies” [2] (Figure 1(d)). The exact composition of pale bodies is unclear, but they are immunoreactive for fibrinogen [10, 65, 73, 74], as well as other acute phase proteins [10] suggesting they contain a mixture of proteins that may reflect a fundamental defect in protein folding or secretion. Hyaline bodies (cytoplasmic inclusions that are eosinophilic and tend to be smaller than pale bodies) are also present in nearly half of FLC cases [2]. Neither pale bodies nor hyaline bodies are unique to FLC, and a diagnosis of FLC should not be made on the presence of these inclusions alone, as they can also be found in ordinary hepatocellular carcinoma. Mild macrovesicular steatosis can be seen in a minority of FLC cases [53]. 

 Mitotic figures are less common in FLC than in usual hepatocellular carcinoma [67]. Cholestasis is often present in FLC [10], with canalicular bile plugging the most common cholestatic pattern. Because of the frequent cholestasis, FLC commonly has copper deposition [2, 10, 69, 73, 75, 76]. Of note, copper accumulation is also common in ordinary HCC that is cholestatic and is not a defining feature of FLC [77]. Microscopic vascular invasion is present in 40–50% of FLC cases [36, 45, 67]. Given the very high rate of recurrence and distant metastasis after surgical resection, it appears very likely that angiolymphatic invasion is even more frequent than these numbers suggest. Occasionally, epithelioid granulomas can be found within the tumor [78] as well as the nonneoplastic liver tissue.

**Figure 2:**
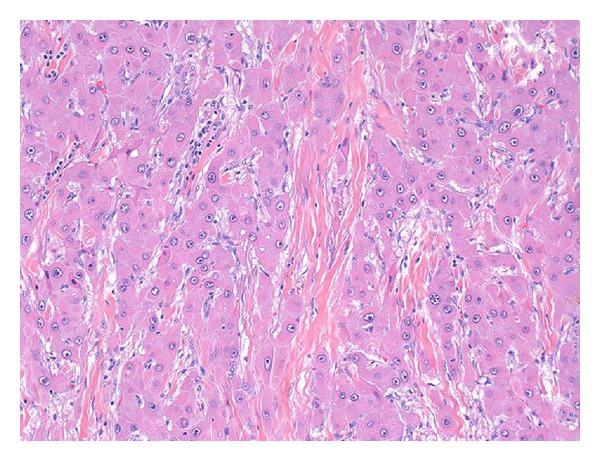
Original magnification 64X. A low power image demonstrates the characteristic lamellar fibrosis.

The tumor typically grows with broad pushing borders, but occasionally benign portal tracts can be found entrapped within the growing front of the tumor. One of the most characteristic low power features of the tumor is the presence of lamellar bands of fibrosis (Figure 2), which are present in essentially all primary tumors and can at times be seen in metastatic deposits. The bands of fibrosis are composed of type I, III, and V collagen and the collagen is produced by the stromal cells within the fibrous bands, although occasional tumor cells also produce collagen mRNA [79]. In about 60–70% of cases, the fibrosis will coalesce into larger central scars with radiating fibrous bands [78]. Calcifications are seen in 68% of FLC by CT studies [72] and small calcifications are not uncommon histologically, usually being seen in the central scar or fibrous bands. 

 In terms of tumor grade, almost all FLCs are moderately differentiated. In fact, it is so unusual for FLC to be poorly differentiated that any poorly differentiated tumor should be carefully examined before making a diagnosis of FLC. Even in recurrent and metastatic FLC, the tumor grades are typically moderately differentiated. 

**Figure 3:**
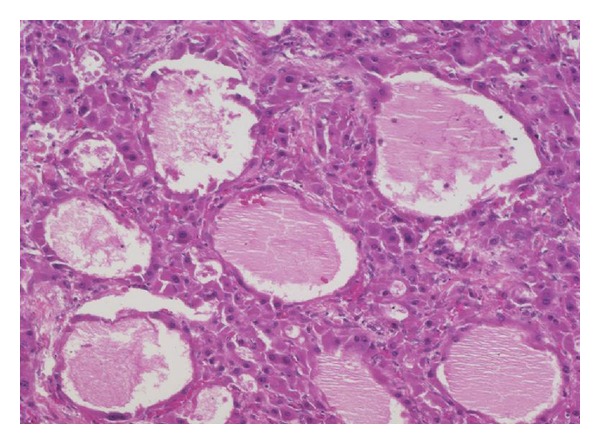
Original magnification 64X. An area of glandular-type differentiation with mucin production is shown.

Of note, some cases of FLC can demonstrate areas of gland-like (pseudoglands) formation with mucin production [3, 80–82]. The pseudoglands are circular to ovoid cystic structures lined by neoplastic cells. The lining cells may be somewhat smaller than the cells in the more typical areas of the tumor, but are otherwise morphologically similar (Figure 3). Mucin can be detected both within individual neoplastic cells as well as in the pseudoglands, and the mucin is positive for mucicarmine in just over half the cases and alcian blue in all cases [80, 82]. The cells lining the pseudoglands express biliary type cytokeratins and other proteins more typical of glandular differentiation. FLC with gland-like areas and mucin production has been called combined FLC and cholangiocarcinoma in the literature, but the best available evidence suggests that they are best considered as typical FLC. There is no strong evidence that they should be classified as true cholangiocarcinomas.

### 3.3. Immunohistochemistry

Immunohistochemical studies of protein expression in FLC have shown the neoplastic cells are strongly HepPar positive [35, 68], even in areas with pseudoglandular differentiation and mucin production [80]. Glypican-3, however, is positive in only a subset of cases, ranging from 17% to 59% of cases [35, 83]. 

The neoplastic cells show expression of the expected hepatocellular cytokeratin 8 [84] and 18 [84], but also are strongly positive for cytokeratin 7 [35, 68, 83–86] and occasionally (between 5 and 25%) for cytokeratin 19 [35, 68, 83, 84]. These latter cytokeratins are normally expressed in biliary epithelium. Immunostains for AFP are negative [35, 60, 69]. The occasional reported cases with AFP positivity are likely misdiagnosed. It remains possible that rare FLC may express AFP, but there have been no convincing images published of AFP producing FLC. Ep-CAM is generally positive [35].

Neuroendocrine features in some FLCs have been noted by several authors [85, 87, 88]. Most FLCs are negative for chromogranin and synaptophysin by immunohistochemistry [35, 69], but occasional cases positive for chromogranin have been reported [23]. Of note, features of neuroendocrine differentiation are also seen in ordinary hepatocellular carcinoma, particularly in those that produce bile, and thus are not unique to FLC [89]. As noted previously, FLCs are also typically bile producing cancers and perhaps the bile production is a common denominator for neuroendocrine features. 

### 3.4. Immunostains to Confirm the Diagnosis of FLC

**Figure 4:**
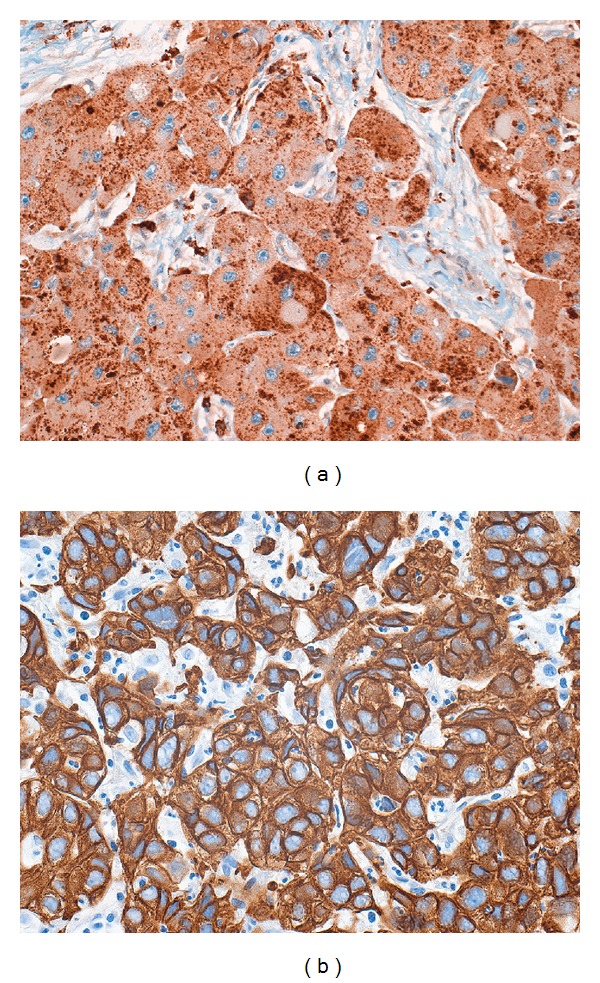
(a) Original magnification 100X. Fibrolamellar carcinomas express CD68 with a characteristic granular cytoplasmic staining pattern. CD68 stains lysosomes within the cytoplasm of the tumor cells. (b) Original magnification 160X. Fibrolamellar carcinomas strongly express cytokeratin 7.

Because some of the cases published in the literature do not appear to truly be FLC, it is clear that the H&E features alone are insufficient to make the diagnosis of FLC with sufficient specificity. The H&E findings appear to be very sensitive, but a significant proportion of typical HCC cases can have some areas that focally resemble FLC. This problem is underscored by reported cases of “mixed hepatocellular carcinoma and FLC” in the literature that are almost certainly not FLC. This problem with the specificity of the H&E findings was formally assessed in one study, which identified significant discrepancies in making the diagnosis of FLC amongst a group of experts in liver pathology [90]. These observations argue strongly for the use of additional tests to confirm the diagnosis of FLC. A recent study has found that FLCs uniformly are positive for CD68 [91]. When this observation is combined with the findings from many other studies that demonstrate that FLCs are strongly positive for CK7, a panel of markers becomes evident. All potential cases of FLC should be stained with CK7 and CD68 to confirm the H&E impression (Figures 4(a) and 4(b)). Those cases that are negative for CK7 and CD68 are most likely not FLC and consideration should be given to send them out for an expert opinion. CK7 and CD68 immunostains should be used together, as a small proportion of typical hepatocellular carcinomas can be CD68 positive [91], and a significant minority of typical hepatocellular carcinomas can also be CK7 positive, in particular hepatocellular carcinomas in children and young adults [68]. Thus, H&E findings should be used as a first screen for the diagnosis of FLC and all cases with H&E findings that suggest the diagnosis of FLC should then be confirmed by immunostaining [91]. At the diagnostic level, additional factors that strongly suggest a diagnosis of FLC may be incorrect include the following: the presence of significant fibrosis in the background liver; AFP elevations in the serum or AFP positivity in the tumor cells by immunostaining; and areas of tumor that lack the key histological features of FLC.

### 3.5. Ultrastructural Findings

On ultrastructural examination, the neoplastic cells show abundant mitochondria [2, 19, 23, 74, 92]. Neurosecretory granules or other evidence of neuroendocrine differentiation can also be seen [23, 92]. Peroxisome-like bodies containing crystalloid material have been reported, along with filamentous material resembling Mallory hyaline [93]. Intracellular lumina, occasionally with bile, have been identified in some but not all cases [93–95]. Interestingly, FLC cells in cell culture may also have intracellular lumina (Figure 1(c)). By electron microscopy, some of the intercellular lumina contain microvilli [95, 96]. The pale bodies appear to correlate with larger endoplasmic vacuoles [74] or intracellular lumina containing numerous microvilli, with the largest ones developing a dense central hyaline core and eventually losing their microvilli [94]. 

### 3.6. Association with Other Liver Tumors

**Figure 5:**
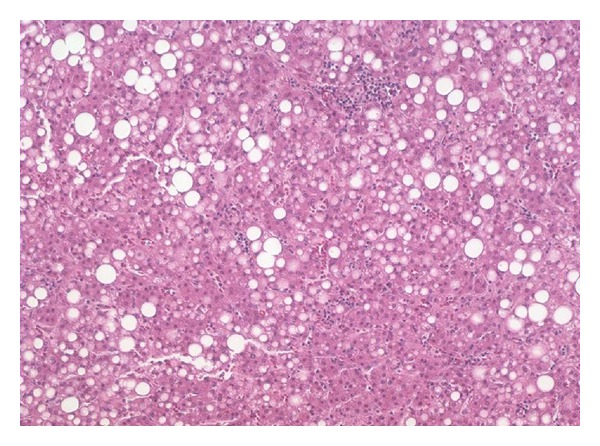
Original magnification 64X. This patient had a typical fibrolamellar carcinoma. Adjacent but clearly separate was a separate nodule of well-differentiated hepatocellular neoplasm with fatty change that lacked the features of fibrolamellar carcinoma.

Rarely FLC and ordinary hepatocellular carcinoma or hepatic adenomas are found in the same liver. In such cases (Figure 5), the two components are typically adjacent but clearly separate [97, 98]. Others have reported FLC with a separate synchronous typical hepatocellular carcinoma [99] or cases in which the recurrence following resection for FLC was a typical hepatocellular carcinoma [100, 101]. In another case, a resected adenoma was followed five years later by an FLC [74]. Stipa et al. reported that 3/41 (7%) cases of FLC had areas resembling conventional hepatocellular carcinoma, but they are not illustrated and the significance and best histological diagnosis is not clear [36]. The intratumoral fibrosis in FLC can vary, and foci with less fibrosis in an otherwise typical FLC should not be misinterpreted as a mixed tumor. 

 FLCs have been also found in association with focal nodular hyperplasia [75, 102, 103]. Because of their shared central scar, copper accumulation, and other features, it was hypothesized that focal nodular hyperplasia may be a precursor lesion to FLC [75, 104]. However, at this time, there is no evidence to support an etiological association, and the nodular hyperplasia that surrounds a small subset of FLC is now recognized as a reaction to the tumor itself and not a precursor.

### 3.7. Tumor Spread

FLCs are locally aggressive and have a high frequency of distant metastases. Based on imaging findings, 42% of FLCs extend outside the liver into the adjacent fat planes [12]. Overall, lymph node and peritoneal metastases are more common in FLC than in typical hepatocellular carcinoma [2]. 

Approximately 50%–70% of individuals with FLC have positive lymph nodes at the time of presentation [7, 12, 36, 66]. FLCs commonly involve regional lymph nodes [23, 45, 92], including the celiac nodes [45, 92], gastric nodes [65], and para-aortic nodes [45, 65]. Direct extension into the stomach [65], diaphragm [12], and pancreas [12, 76] have all been reported. Metastatic disease to intra-abdominal organs, as well as peritoneal spread, is common [12, 19, 36, 45, 66, 71, 96]. Pulmonary metastases are frequently reported [11, 37, 45, 65, 71], and adrenal metastases are also common [12]. Rare metastatic sites include bone [11, 71] and ovary [105]. 

**Figure 6:**
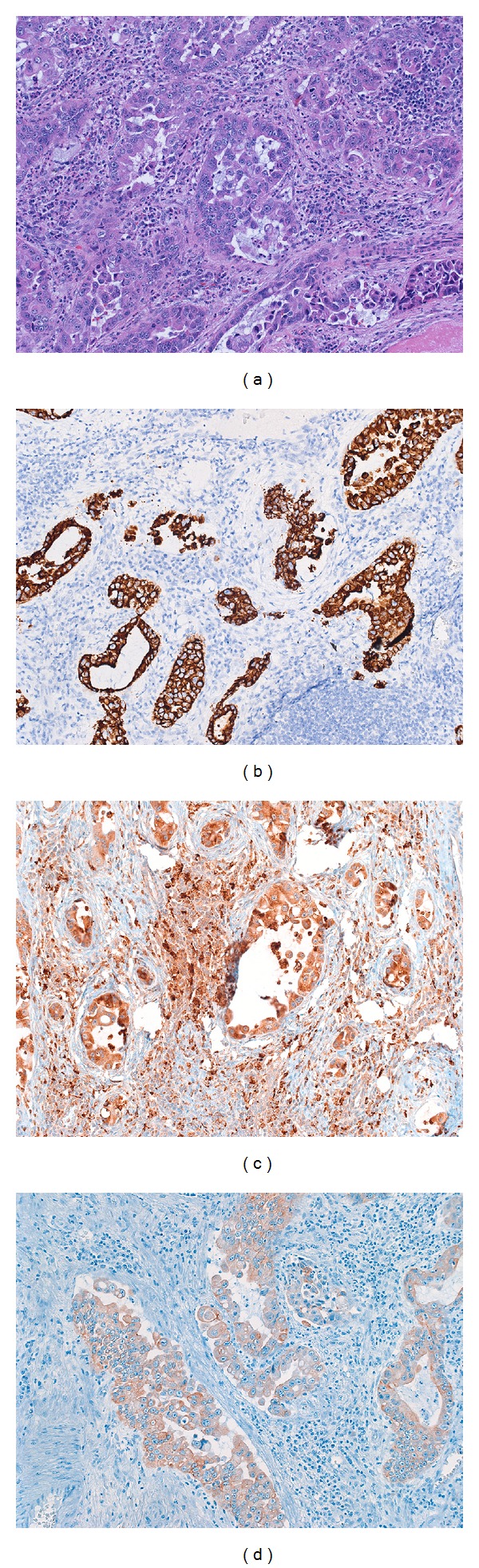
Original magnification 64X. A patient with a typical fibrolamellar carcinoma had a subsequent lymph node metastasis. The metastatic carcinoma had a distinctive glandular growth pattern that mimicked a cholangiocarcinoma. However, the metastatic tumor was still HepPar positive ((b), original magnification 64X), CD68 positive ((c), original magnification 64X). Cytokeratin 7 was also strongly positive (not shown), while cytokeratin AE1/3 was weakly positive ((d), original magnification 64X).

The distinctive tumor cytological findings (large pink cells with prominent nucleoli) are seen in most metastatic deposits. In addition, the distinctive lamellar fibrosis can also be seen in cases of metastatic disease [2, 105]. Because of these findings, most cases of FLC are readily diagnosed from metastatic sites. Rarely, tumor metastases can have a predominately glandular morphology (Figures 6(a)–6(d)), including mucin production, and be mistaken for a second primary. Immunostains can be very helpful in making the diagnosis of metastatic FLC (Figures 6(a)–6(d)).

### 3.8. Section Summary and Key Questions

The gross and histological findings of FLC have been well studied, and the key findings are well established. These findings include tumor cells with abundant cytoplasm, large nucleoli, and extensive intratumoral fibrosis that can often coalesce into a central scar. While the H&E findings are distinctive, history has shown that they are not sufficiently specific across different study centers and immunohistochemical studies (CD68 and CK7) should be used to confirm the diagnosis. This is important to ensure that data results are accurate and can be compared across different study centers. Other immunohistochemical-based studies have been useful in highlighting the unique differentiation of FLC tumor cells—they clearly express features of hepatocytes, biliary cells, and neuroendocrine cells. 

Key questions that remain to be answered or substantially clarified include the following: *Key Question *1. What is the cell of origin of FLC? If a normal counterpart of FLC can be identified in the developing or mature liver, this may give important clues to etiology and to potential treatments. *Key Question *2. While AFPs producing FLCs have been reported, it remains unclear if they are actually misclassified hepatocellular carcinoma. If there are truly AFP producing FLC, do they differ in clinical findings, histological findings, or prognosis? *Key Question *3. In a similar fashion, the best classification of the reported cases of “mixed FLC and typical hepatocellular carcinoma” remains to be determined to clarify if this entity truly exists or if they are misclassified and are not actually FLCs. It seems clear from a few reported cases that FLC can very rarely coexist with a separate nodule of a typical hepatocellular neoplasm (see also Figure 5), but this phenomenon appears to be much more rare and quite different than the “mixed” tumors that have been reported by others. 

## 4. Etiology

The etiology of FLC is unknown. Interestingly, FLCs are also reported to occur in other mammals and so are not unique to humans [106]. FLCs do not generally arise in the setting of any known chronic liver disease: the background livers typically lack significant inflammation or fibrosis [68]. While occasional reports have described the presence of hepatitis B viral proteins or DNA in FLC [2, 61, 107, 108], this appears to be by chance given the high worldwide prevalence of chronic hepatitis B infection and there is no data to suggest hepatitis B as an etiological agent. Likewise, FLCs have also developed in women using oral contraceptive pills [53, 109], but the association again appears to be by chance. No histological precursor lesion to FLC has been identified. In one study, occasional foci of altered hepatocytes were found in the background livers of resected FLC specimens [68], but the small number of cases and the fact that the background livers were not extensively sampled limits interpretation. Rare cases of FLC have been reported to arise in association with other malignancies or in the setting of well-defined inherited syndromes (Table 3). While FLCs are clearly not a major component of any of these inherited syndromes, their occurrence in these patients suggest the possibility of involvement by shared molecular pathways.

## 5. Molecular Findings

The molecular basis for FLC remains largely unknown. Only a limited number of individual oncogenes and signaling pathways have been studied in FLC, but the results indicate that the uniqueness of FLC extends to the molecular level. As a caveat, a proportion of cases included in this literature may not be FLC, so the data should be interpreted with some caution.

### 5.1. Large Scale DNA Changes

DNA ploidy studies have shown that half of the FLC cases are aneuploid [74, 112] while the other half are tetraploid [112]. Despite these ploidy abnormalities, the tumor appears to be relatively chromosomally stable with few large chromosomal changes, compared to typical hepatocellular carcinoma [113], though recurrent FLC or metastases may show increased chromosomal abnormalities [114, 115]. Overall, the available data suggests the possibility of several recurrent cytogenetic abnormalities. For example, in one cytogenetic study of ten FLCs, three cases had no cytogenetic abnormalities, six cases had gains of chromosome 1q, and three cases had loss of chromosome 8p [113]. Others have also reported chromosome 1q abnormalities [15, 114]. The many cytogenetic studies on FLC are nicely summarized in the review article by Ward and Waxman [5]. The chromosomal stability of FLC is further supported by finding infrequent allelic loss using DNA probes [116]. Genomic homogeneity of FLC was also found by arbitrarily primed PCR [117]. 

### 5.2. Microsatellite Instability

Microsatellites are small segments of repetitive DNA that are present in normal genomic DNA. However, mutations in DNA repair genes can lead to widespread mutations, including changes in the length of the microsatellite DNA fragments. These mutations can lead to carcinoma. We studied three FLCs and found no microsatellite instability (unpublished observations).

### 5.3. Specific Mutations

#### 5.3.1. TP53

While p53 protein is over-expressed in approximately 25% of typical HCC in the USA [118], it is only rarely over-expressed in FLC [119] and no *TP53* mutations were found in FLC by denaturing gradient gel electrophoresis [120]. 

#### 5.3.2. Beta Catenin

Mutations or overexpression of the beta catenin gene (*CTNNB1*), a key member of the Wnt signaling pathway, have been found in all other categories of hepatocellular neoplasms including hepatoblastomas, hepatic adenomas, and typical hepatocellular carcinoma [121]. While no mutations are found in FLC [122], and there is no nuclear accumulation of beta catenin by immunostain, there is some evidence that the Wnt signaling pathway may still be active in FLC [123].

### 5.4. Mitochondrial DNA Changes

As discussed previously, FLCs are characterized by increased mitochondria. However, careful analysis of the DNA content of the mitochondria found that the tumor cells are actually relatively depleted of mitochondrial DNA [124]. Complete sequencing of the mitochondrial DNA found no consistent mutation pattern to explain these changes in mitochondrial DNA [124].

### 5.5. Epigenetic Changes

Hepatocellular carcinomas frequently have hypermethylation of the promoters of tumor suppressor genes. FLCs also have abnormally methylated tumor suppressor gene promoters [125, 126], but the overall levels of hypermethylation are less than that of typical hepatocellular carcinomas arising in cirrhotic livers and more similar to those of hepatocellular carcinomas arising in noncirrhotic livers [125]. 

### 5.6. Specific Over-Expressed Pathways 

#### 5.6.1. Aromatase

The tumor cells of FLC overexpress aromatase [14, 15, 127] and individuals with FLC may present clinically with gynecomastia (Table 1). High serum estrone and estradiol levels are also found in these cases at presentation [14, 15]. Aromatase converts androgens to estrogens, more specifically androstenedione to estrone and testosterone to estradiol. Aromatase expression is under the control of several different tissue specific promoters. Aromatase levels are high in the developing liver but disappears in the postnatal liver. Interestingly, abnormal aromatase expression in FLC was not linked to abnormal activation of the fetal liver promoter, but instead to activation of promoters more commonly used in various endocrine cells [14]. 

#### 5.6.2. Neurotensin

Other proteins that can also be expressed in FLC [128] but are normally restricted to the fetal liver include neurotensin [129], an endocrine agent.

#### 5.6.3. mTOR

The mTOR pathway, important in energy metabolism and protein translation, is activated in 47% of FLCs, a finding similar to typical hepatocellular carcinoma [130]. 

#### 5.6.4. EGFR

 Epidermal growth factor receptor (EGFR) is over-expressed in the majority of FLCs by immunostaining [131, 132] as a result of polysomy of chromosome 7 [132].

#### 5.6.5. AGR2 

**Figure 7:**
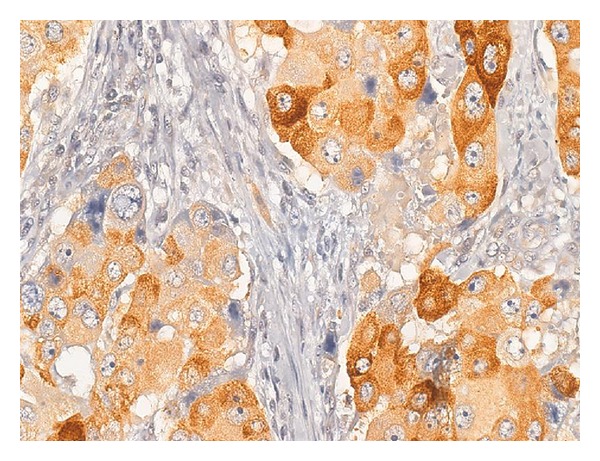
(Original magnification 100X). Fibrolamellar carcinomas are strongly AGR2 positive.

FLCs, but not typical hepatocellular carcinomas, also strongly overexpress anterior gradient 2 (Figure 7), [133]. Anterior gradient 2 is a protein normally expressed in the developing fetal gastrointestinal tract. In the fetal liver, anterior gradient 2 is expressed in the large size bile ducts and the gallbladder but not the smaller sized bile ducts in the periphery of the liver [134]. In the postnatal liver, anterior gradient 2 continues to be expressed in the gallbladder [134] and large intrahepatic bile ducts [133, 134]. Also of note, anterior gradient 2 is expressed in the postnatal gut, where it has an important role in regulating neuroendocrine cell specific lineage in the gastrointestinal tract and is found in all neuroendocrine cells that produce chromogranin as well as goblet cells [135]. 

#### 5.6.6. Miscellaneous

A wide variety of other proteins have been studied (Table 4). Despite the common overexpression of the antiapoptotic gene survivin in typical HCC, no [119] or limited [83] overexpression was found in FLC. Other studies have shown that the hepatocyte growth factor [136] and plasmin systems [136] as well as TFF-beta [137] are more frequently activated in HCC than in FLC. 

The tumors show overexpression of matrix metalloproteinase 2 [136] and the matrix itself shows increased expression of tenascin but limited expression of basement membrane components [142]. 

### 5.7. Section Summary and Key Questions

 FLCs are relative chromosomal stable with frequent gains of chromosome 1q. They have epigenetic changes, but not as many as those found in typical hepatocellular carcinomas in cirrhotic livers. They lack mutations that are commonly found in typical hepatocellular carcinoma, such as *CTNNB1* and *TP53*. FLCs do not have consistent or frequent mitochondrial mutations but do appear to have mitochondria biogenesis defects. Large numbers of lysosomes are found in their cytoplasm, suggesting a potential role for autophagy in tumorgenesis. 

In keeping up with the histological findings discussed in the previous section, molecular studies confirm key aspects of neuroendocrine differentiation. FLCs frequently overexpress aromatase, perhaps through the use of a neuroendocrine specific promoter. 

FLCs also show overexpression of the EGFR and the mTOR pathways, both pathways which are also commonly over-expressed in typically hepatocellular carcinomas and may have therapeutic possibilities. 

Key questions that remain to be answered or substantially clarified include the following: *Key Question *1. The most important question in this section is: what are the genetic changes that drive the formation of FLC? The typical homogenous appearance of FLC suggests that tumor formation may be largely driven by a relatively small number of key changes/mutations in a single pathway. Their unique age at presentation and the extensive histological data indicate that the tumors do not result from extended levels of low but persistent DNA damage due to chronic liver disease. They also do not have the classic pattern of neonatal tumor formation that can be seen in many cases of cancers resulting from inherited mutations or mutations acquired in utero (e.g., retinoblastoma and hepatoblastoma). There is no evidence of microsatellite instability, and they do not have unusually high levels of epigenetic instability. Nor do they manifest the “field effect” that can be seen in other forms of inherited cancer that can present in early adult years (e.g., colon cancer in the setting of familial adenomatosis polyposis). Thus, the genetic changes that lead to FLC appear to be relatively unique and their discovery may provide fundamental new insights into tumor genesis in general. *Key Question *2. Since recurrent and metastatic disease is such a major problem after liver surgery, studies are needed that examine the genetic and epigenetic changes in primary versus metastatic disease. While a few studies have included metastatic tumors [124, 127], there is very little data on this topic. Yet, there could be important difference that would be relevant to chemotherapy treatments. *Key Question *3. Critical basic science reagents are needed to advance the field, in particular robust cell lines and animal models.

## 6. Conclusions

FLCs are unique at the clinical, histological, and molecular levels. They are primary cancers of mixed differentiation (hepatocellular, biliary, and neuroendocrine) that occur in young individuals with no known liver disease and no precursor lesions. Their etiology is unknown and much of their molecular biology remains poorly described and awaits future investigation. FLCs are aggressive tumors with an overall low cure rate. Yet, there is hope that improvements in therapy will develop as advanced molecular biology tools are applied to the field and uncover the principle genetic lesions that drive tumor growth.

## Figures and Tables

**Table 1:** Unusual presentations for fibrolamellar carcinoma.

Category	Presentation
Vascular flow abnormalities	Budd-Chiari [17, 18]
	Caval compression syndrome [19]
	Caval obstruction [20]

Systemic symptoms	Severe anemia hypoglycemia [21]
	Increased beta hCG [22]
	Systemic AA amyloid deposition [23]

Biliary	Obstructive jaundice [24, 25]
	Growth into bile duct [26]
	Background disease of ulcerative colitis and primary sclerosing cholangitis [27]

Miscellaneous	Nonbacterial thrombotic endocarditis [28]
	Following autologous bone marrow transplant for lymphoma
	Extensive intraperitoneal metastases with ascites [29]
	Mimicking a liver abscess [30]
	Bone metastases [31]
	Gynecomastia [13–15]
	Pancreas tumor with liver metastasis [32]
	Cold agglutinin disease [33]

**Table 2:** Representative studies of the prevalence of FLC in various populations.

Location	Prevalence	Comment
Ostra Sjukhuset, Sweden [63]	2/532 (0.4%)	Based on cases from 1958 to 1979
Songkhla, Thailand [64]	1/180 (0.6%)	Southern Thailand, from 1991 to 1998
USA [47]	71/9,870 (0.7%)	From SEER database, based on cases from 1986 to 2000
Columbus, OH, USA [65]	3/58 (5%)	Ohio State, cases from 1966 to 1981
Rochester, MN, USA [66]	10/280 (4%)	Mayo clinic, cases from 1987 to 1993
Pittsburgh, PA, USA [45]	41/477 (8.9%)	Patients undergoing liver transplant for HCC between 1968 and 1995
Rochester, MN, USA [66]	10/280 (4%)	Mayo clinic, cases from 1987 to 1993
Mexico City, Mexico [34]	7/121 (5.8%)	Based on cases from 1987 to 2001
Mexico City, Mexico [53]	15/174 (8.6%)	Based on cases from 1990 to 2003
Villejuif, France [52]	5/68 (7.3%)	Frequency of HCCs in noncirrhotic livers
South Africa [54]	9/274 (3%)	Data from pediatric cancer registry; includes all pediatric cancers and not just HCC
London, UK [39]	8/107 (7%)	
Toronto, Canada [11]	10/NS (9%)	Referrals to a tertiary care center for HCC with intent to treat from 1982 to 1995

**Table 3:** Reports of FLC in the setting of inherited syndromes or with other nonhepatic tumors.

Syndrome	
Wilms [51]	
Carney [74]	
Fanconi anemia [110]	
FAP [111]	

**Table 4:** Protein expression changes in FLC.

Protein	Observation
Endothelin-1 and 3	Overexpressed [138]
EGFR	Overexpressed [131, 132]
TGF-*β*	Overexpressed [137]
NF-*κ*B	Overexpressed [139]
p16	Overexpressed [140]
SHP	Underexpressed [141]
